# 5-Fluorouracil-Induced Acute Toxic Leukoencephalopathy: A Rare Adverse Effect and Its Early Detection on MRI

**DOI:** 10.7759/cureus.98058

**Published:** 2025-11-29

**Authors:** Ambar Ambreen, Muhammad Atif Naveed, Khalid Javed, Muhammad Qasim Naeem, Nahel Chaudhry

**Affiliations:** 1 Radiology, Shaukat Khanum Memorial Cancer Hospital and Research Centre, Lahore, PAK; 2 Medical Oncology, Shaukat Khanum Memorial Cancer Hospital and Research Centre, Lahore, PAK

**Keywords:** 5-fu(5-fluorouracil, (capeox), diffusion weighted imaging (dwi), mri, toxic leukoencephalopathy

## Abstract

This case report discusses a rare occurrence of 5-fluorouracil (5-FU)-induced encephalopathy, an adverse effect of this commonly used antimetabolite and antineoplastic agent. While 5-FU is primarily associated with systemic side effects such as gastrointestinal issues and neutropenia, its cerebral involvement is infrequently documented. Diagnostic imaging techniques, such as computed tomography (CT) and magnetic resonance imaging (MRI), are essential for diagnosing and monitoring this condition. Early recognition and treatment are crucial for a favorable prognosis. We present a case of 5-FU-induced encephalopathy, which was successfully diagnosed based on clinical and MRI findings.

## Introduction

5-fluorouracil (5-FU) is a common chemotherapy drug used to treat various cancers of the digestive tract. It acts as a thymidylate synthase inhibitor and hinders the process of DNA replication [1]. While it is generally not associated with neurotoxicity, when that does occur, it can manifest either acutely or with a delayed onset. The incidence of 5-FU-induced leukoencephalopathy is less than 5% among patients treated with this drug [2].

Toxic leukoencephalopathy (TL) is a central nervous system disorder primarily affecting the white matter. It has various causes including radiotherapy to the cranium, chemotherapy, exposure to toxins and recreational drug usage [3]. TL can present with a wide range of clinical manifestations, from mild cognitive deficits that may be mistaken for psychiatric disorders to profound neurological impairment [4]. During the acute phase, acute TL may exhibit a distinct and significant MR imaging appearance, which has the potential to reverse with appropriate treatment or discontinuation of the causative agent [4].

This case report details a rare example of a patient who developed acute neurotoxicity following a continuous infusion of 5-FU during systemic chemotherapy, and highlights the successful early diagnosis of this condition at a specialized cancer treatment center.

## Case presentation

**Figure 1:**
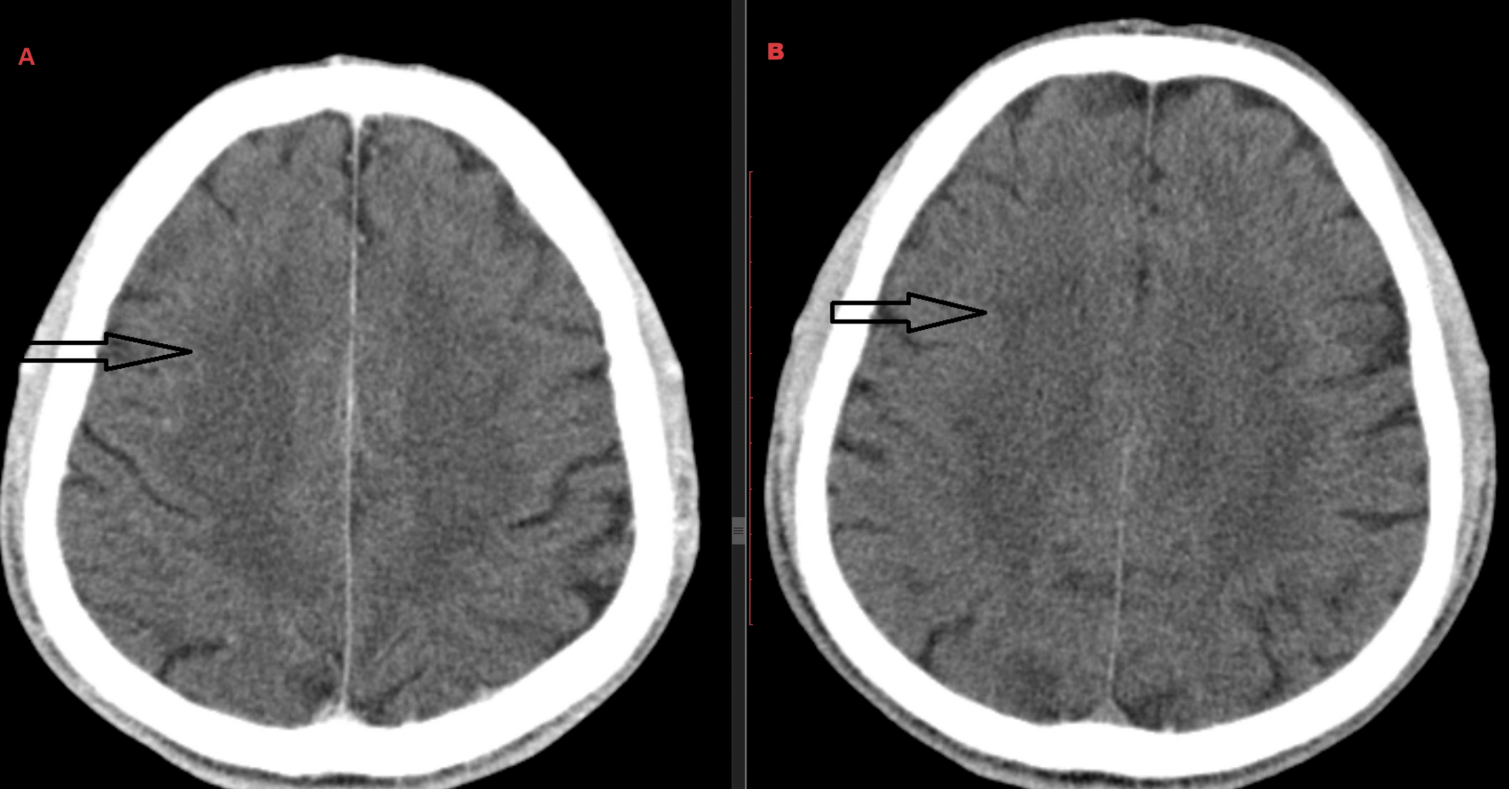
Initial CT brain plain (December 2023) shows no abnormality.

A 25-year-old male with history of adenocarcinoma of the colon in 2019 underwent right hemicolectomy and received adjuvant chemotherapy with capecitabine and oxaliplatin (CapeOx), completing eight cycles. Follow-up was intermittent due to missed appointments by the patient, and in July 2023, he presented with disease progression for which he was started on FOLFOX chemotherapy (folinic acid, 5-FU, and oxaliplatin) for approximately 10-12 cycles. During the second cycle, the patient complained of headache. A plain CT brain was ordered but was unremarkable (Figure 1).

Following the third cycle, the patient presented to the ER with severe headaches and was managed with analgesics. These symptoms worsened upon the completion of the 11th cycle, when the patient exhibited dizziness and slurred speech in addition to the severe headache. An urgent CT of the head was again unremarkable; the patient was managed conservatively and discharged.

**Figure 2:**
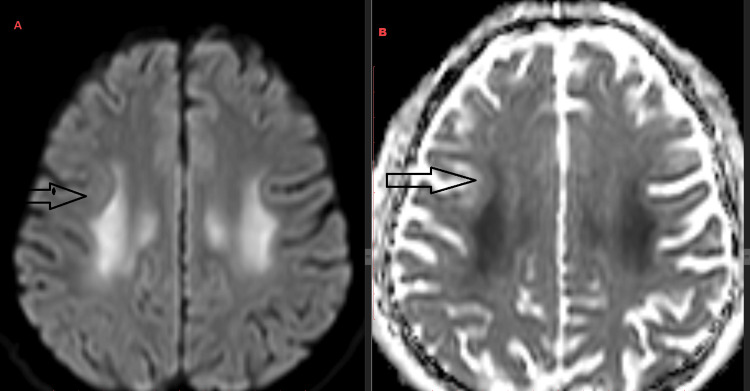
MRI diffusion-weighted imaging (DWI) (a) and apparent diffusion coefficient (ADC) (b) images from December 2023; shows bilateral symmetrical periventricular white matter diffusion restriction.

After the 12th cycle, the neurological symptoms returned and an MRI brain was requested. The scan showed symmetrical decreased diffusivity in the centrum semiovale, corona radiata, splenium of the corpus callosum, and cerebral peduncles of the midbrain (Figure 2). At this point, the FOLFOX chemotherapy was discontinued, oxaliplatin was excluded from the regimen, and chemotherapy with FOLFIRI (irinotecan, folinic acid, 5-fluorouracil) was suggested.

**Figure 3:**
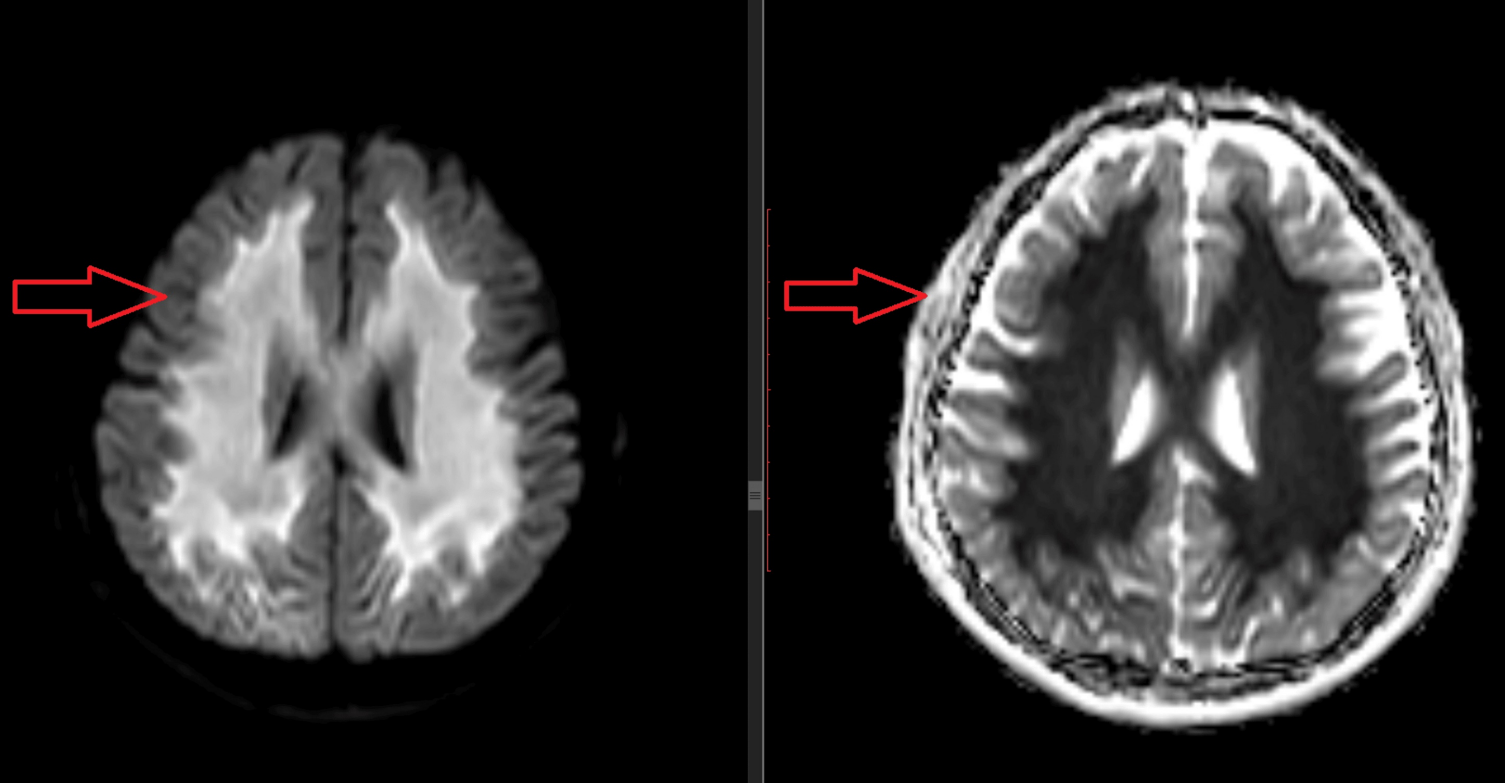
MRI diffusion-weighted imaging (DWI) (a) and apparent diffusion coefficient (ADC) (b) images (March 2024) shows progressive disease with increase in bilateral symmetrical periventricular white matter areas of diffusion restriction.

After one cycle of FOLFIRI, the patient developed abnormal movements and hand twisting, and a diagnosis of chemotherapy-induced leukoencephalopathy was made. Chemotherapy was withheld and a repeat MRI brain was ordered which revealed worsening of the previously noted findings (Figure 3).

The patient developed seizures and was managed with anticonvulsants and intravenous steroids. Upon improvement of neurological symptoms, he was discharged with oral steroids. Further chemotherapy continued with single-agent irinotecan for four cycles.

## Discussion

Toxic leukoencephalopathy (TL) is primarily caused by various chemotherapeutic agents, such as methotrexate, vincristine, ifosfamide, fludarabine, cytarabine, 5-fluorouracil, cisplatin, and interferons [5]. Factors that increase the risk of developing TL include being female and having liver insufficiency [6]. The chemotherapy drug 5-FU, used commonly in cancer treatment, causes neurotoxicity in fewer than 5% of patients. 5-FU works by blocking DNA synthesis through the reduction of thymidine monophosphate formation, inhibiting thymidylate synthase, and integrating into RNA. It effectively crosses the blood-brain barrier [5].

The exact pathophysiology of TL is not fully understood. However, research indicates that the initial phase involves damage to the myelin sheath, formation of vacuoles in brain tissue, swelling of myelin, and infiltration by immune cells (macrophages), which restricts water movement. This process explains the abnormal signals seen on diffusion-weighted MRI scans due to fluid buildup from toxic swelling [7].

Imaging is essential for visualizing lesions, their locations, and their extent. While CT scans may be normal or inconclusive, MRI provides superior resolution and can pick up lesions missed by CT scans. Diffusion-weighted imaging (DWI) and apparent diffusion coefficient (ADC) maps can reveal lesions due to edema within intramyelinic clefts, which may resolve once the toxic agent is removed. Typically, the deep white matter and corpus callosum are affected, while the basal ganglia, thalamus, and U-fibers are generally spared [8].

Drug-induced leukoencephalopathy should be considered in patients who are receiving chemotherapeutic agents, especially 5-FU, and showing altered consciousness or abnormal neurological findings. Prompt MRI, particularly diffusion-weighted imaging, is crucial for confirming the diagnosis [9].

Differential diagnoses for acute TL include posterior reversible encephalopathy syndrome (PRES) and radiation-induced angiopathy. PRES, often triggered by drugs such as cyclosporine, tacrolimus, and interferon alfa, initially affects the cortex and subcortical white matter, with progression to periventricular white matter in severe cases. Diffusion restriction may be observed in a minority of cases. Radiation injury typically presents as small vessel ischemic demyelination, characterized by asymmetrical hyperintensity on fluid-attenuated inversion recovery (FLAIR) in the periventricular deep white matter without diffusion restriction [8].

Other differential diagnoses, like carbon monoxide poisoning and heroin-induced leukoencephalopathy, exhibit similar diffusion restriction in acute phases but have distinct imaging patterns. Carbon monoxide poisoning initially affects deep white matter and later involves deep gray matter, while heroin-induced leukoencephalopathy shows symmetric involvement of cerebral and cerebellar white matter and deep gray matter. Detailed clinical history can distinguish between these conditions [8].

Therapeutic management mainly involves discontinuing the causative drug. Corticosteroids, antioxidants, thiamine infusion, and plasma exchange may be prescribed. Uridine triacetate, a 5-FU antidote, can also be administered within the first 96 hours if available [6].

## Conclusions

5-FU-induced TL poses a significant diagnostic challenge because of its rarity and variable clinical presentation. Accurate identification of the distribution and pattern of MRI abnormalities by the radiologist is essential for prompt diagnosis. Equally important is the contribution of the primary physician, who must provide a thorough clinical history, as many toxic substances present with similar imaging features. Early detection and discontinuation of the offending drug can lead to significant clinical improvement. For effective management, both radiologists and clinicians need to be aware of this condition and perform thorough correlations in multidisciplinary meetings.
